# Methodological limitations of a transdiagnostic study on postpartum depression

**DOI:** 10.1038/s41398-021-01413-8

**Published:** 2021-05-17

**Authors:** Fama Tounkara, Ram P. Sapkota, Alain Brunet

**Affiliations:** 1 Department of Neurosciences and Montreal Neurological Institute-Hospital, McGill University, Montreal, QC Canada; 2 Department of Psychiatry and Douglas Mental Health University Institute, McGill University, Montreal, QC Canada

**Keywords:** Physiology, Human behaviour

Dear Editor,

The paper “Delayed sleep timing and circadian rhythms in pregnancy and transdiagnostic symptoms associated with postpartum depression” by Obeysekare et al.^1^ was both scholarly and interesting. The authors report that delayed sleep timing of pregnant women is associated with psychiatric symptoms, such as depression, obsessive-compulsive disorder, and mania. They argue that sleep timing is a modifiable risk factor for postpartum depression, an encouraging finding from a public mental health perspective. Nevertheless, we wish to point to some methodological issues that make the results difficult to interpret.

First, majority of the study participants (70%) reported a history of trauma exposure. Studies^2,3^ suggest that disturbances in sleep initiation, sleep duration, and waking time after falling asleep occur in individuals suffering from post-traumatic stress disorder. Since trauma exposure was not statistically controlled in the analysis, it remains unclear if the results could have been explained by the women’s traumatic stress, resulting from traumatic experiences.

Second, the authors used two different brands for the wrist actigraph to record the participants’ sleep. In a study that compares brands of actigraphic devices, Meltzer et al.^4^ reported that inter-device reliability was low, and advised against the use of different brands in the same study. Otherwise, it can truly alter the actual data and lead to misinterpretation of the results.

Third, the authors do not provide a rationale for some of their methodological choices. For example, why four measurement points for sleep and only two points for dim light melatonin onset were used? Why the authors chose to measure obsessive-compulsive disorder and exclude anxiety disorders among the measured psychiatric symptoms? An explanation of the choices would have allowed for a better understanding and comparison with the literature.

Fourth, since melatonin levels are extremely sun sensitive^5^, the geographical location, season (and weather) for each participant should have been considered. Indeed, omitting these variables in the study could have impacted the accuracy of the results obtained. Further, multiple tests conducted by the authors may have unduly inflated the alpha level and increased the risk of false-positive findings. Such a risk could have been controlled by using Benjamini’s and Hochberg’s false discovery rate^6^.

In summary, we found this study very interesting, but in the end our enthusiasm was dampened by the study limitations.
